# Management of a unique hourglass-shaped abdominal aortic aneurysms: A case report

**DOI:** 10.1016/j.ijscr.2025.111383

**Published:** 2025-05-03

**Authors:** Javad Salimi, Pejman Pourazari, Afrooz Sadeghi, Amir Shokri

**Affiliations:** Department of Vascular Surgery, Sina Hospital, Tehran University of Medical Science, Tehran, Iran

**Keywords:** Abdominal aorta, Aortic aneurysm, Hourglass-shaped, Surgical procedures, Case report

## Abstract

**Introduction:**

Hourglass-shaped abdominal aortic aneurysms (AAAs) represent a complex variant of aneurysms, characterized by a distinctive constriction within the aorta, which are characteristically located in the abdominal or thoracic regions. Their unique morphology poses significant diagnostic and therapeutic challenges, particularly when considering the feasibility of endovascular repair versus open surgical intervention, demanding a specific approach to management.

**Case presentation:**

We present the case of a 68-year-old female patient admitted with abdominal pain complaints. Despite stable vital signs, her medical history, including diabetes mellitus and reduced ejection fraction (EF) of 40 %, indicated a heightened cardiovascular risk. Subsequent imaging studies, including Doppler ultrasonography and CT angiography, showed the presence of two adjacent abdominal aortic aneurysms forming an hourglass-shaped configuration. Due to the inadequacy of the proximal landing zone for an endovascular procedure, an open surgical intervention was considered necessary to address the aneurysms.

**Discussion:**

Managing the hourglass-shaped AAAs requires careful consideration due to their complex morphology, which can complicate the option between endovascular and open surgical repair. While Endovascular Aneurysm Repair (EVAR) is often a preferred method for high-risk patients due to its minimally invasive nature, in this case, insufficient proximal landing zones made the EVAR unsuitable. Open surgery, although more aggressive, was required to prevent complications such as ensuring a safe repair and rupture. Perspective imaging and patient-specific factors played an important role in guiding the treatment plan, highlighting the importance of individual approaches in managing complex vascular discrepancies.

**Conclusion:**

The present article reports the management of a patient with an hourglass-shaped AAA, emphasizing its relevant diagnostic challenges, the rationale behind treatment decisions, and the outcomes we achieved. Our findings highlight the need for personalized approaches in managing complex aneurysmal presentations.

## Highlights

• Hourglass-shaped abdominal aortic aneurysms (AAAs) are rare and pose unique diagnostic and therapeutic challenges.
• This case report details the successful open surgical repair of a complex hourglass-shaped AAA in a high-risk patient.
• Endovascular aneurysm repair (EVAR) was deemed unsuitable due to insufficient proximal landing zones.
• Preoperative imaging played a crucial role in surgical planning and decision-making.
• This case emphasizes the need for individualized management strategies for complex vascular anomalies.

## List of abbreviations

AAA: Abdominal Aortic Aneurysm
CT: Computed Tomography
CTA: Computed Tomography Angiography
EF: Ejection Fraction
EVAR: Endovascular Aneurysm Repair

## 1 Background

Hourglass-shaped abdominal aortic aneurysms (AAAs) constitute a rare and interesting variant of aortic pathology, which accounts for around 1.3 % of all deaths among men aged 65 to 85 years in developed countries, characterized by a distinct narrowing of the aorta that complicates traditional management approaches. An aortic aneurysm is essentially an abnormal aorta dilation resembling a hernia, which may occur in either the abdominal or thoracic regions (1,2). An aneurysm occurs when the aorta's diameter is more than 1.5 times its normal size (3). These vascular anomalies pose significant risks, including rupture and thrombosis, which can lead to life-threatening situations. Effectively managing AAAs involves thorough evaluation and customized intervention strategies that determine the specific shape of the aneurysm and the patient's overall health. Most individuals with aortic aneurysms remain asymptomatic until a rupture occurs, at which point they may experience severe abdominal and lower back pain, with some severe drop in their blood pressure (4,5).

Early diagnosis and intervention are crucial, as the weakened aorta wall can rupture, leading to catastrophic hemorrhage from this major artery (6). The mortality rate associated with abdominal aortic dissection is reported to be as high as 81 %, with 32 % of patients dying before reaching medical facilities (7).

While abdominal aortic aneurysms are more common than their thoracic counterparts, diagnosing them may appear challenging (1). Although abdominal ultrasound is often used as an initial screening tool, CT angiography remains the gold standard for confirming the diagnosis and plays a crucial role in the detailed evaluation of aneurysm morphology guiding the treatment strategy. These imaging modalities are essential in determining whether a patient should undergo open surgery or an endovascular aneurysm repair (8,9).

In this case report, we present an instance of two adjacent abdominal aortic aneurysms manifested in an hourglass shape. This unique presentation not only posed diagnostic challenges but also required careful consideration in our treatment decisions. The current report gave details of the management of this rare position, which emphasized the diagnosis, treatment strategies, and the complications involved in the results obtained by us. By sharing our findings, we aim to enrich the existing literature on abdominal aortic aneurysms and refine relevant management protocols for this complex condition, ultimately enhancing patient care and outcomes.

## 2 Case presentation

**Fig. 1:**
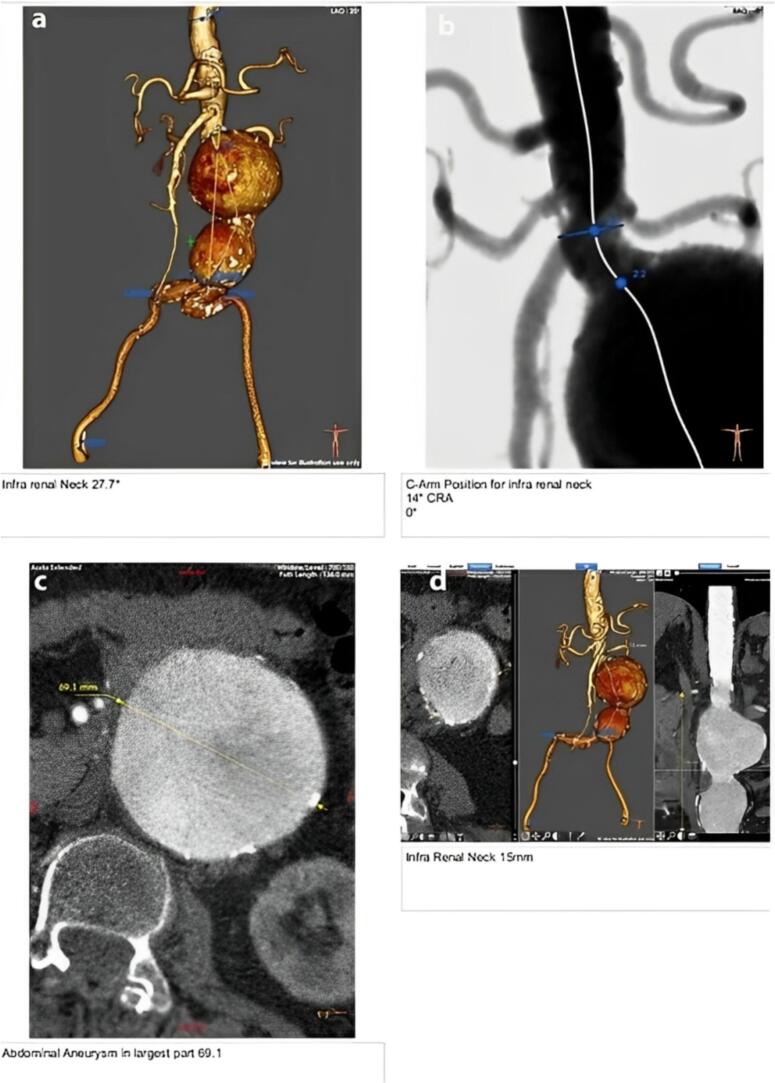
Various Viewpoints of the Aneurysm, including (a) a 3D CT angiographic reconstruction showing an infrarenal abdominal aortic aneurysm with a neck length of 27.7 mm; (b) a fluoroscopic image with optimal C-arm positioning (14° cranial angulation, 0° rotation) for assessment oh = f the infrarenal neck during endovascular planning; (c) an axial CT image demonstrating the maximal transverse diameter of the aneurysmal sac; and (d) #d post-processing and coronal CT images showing an infrarenal neck length of 15 mm, providing crucial information for EVAR feasibility and endograft selection.

A 68-year-old woman was admitted to the emergency department with complaints of persistent abdominal pain over several days. Her vital signs were stable upon her arrival, indicating no immediate hemodynamic instability. A fully physical examination ordered the doctor to Doppler ultrasonography, due to which AAA was diagnosed. To collect more detailed information about the form, size, and location of the aneurysm, a CT angiogram was also done (Fig. 1). This case was reported in line with the SCARE criteria (10).

**Fig. 2:**
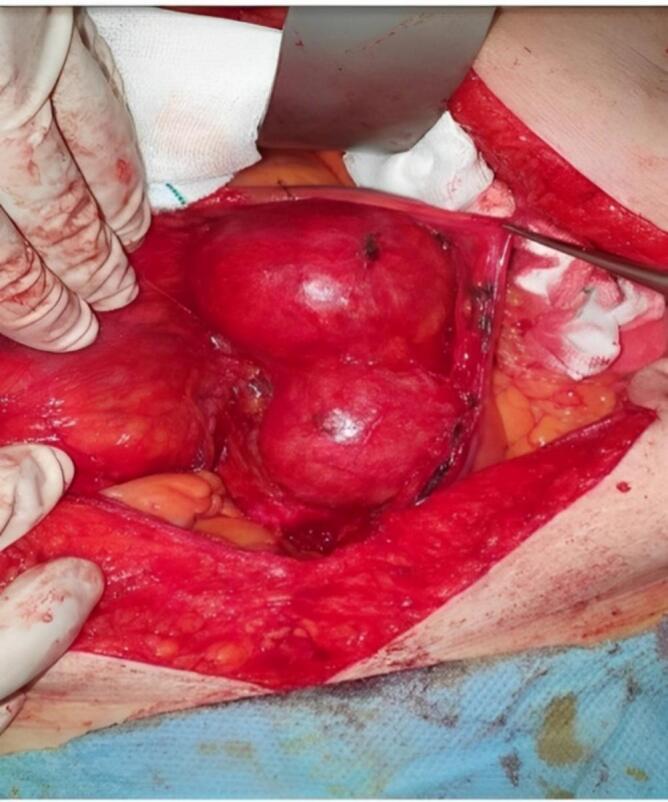
Intraoperative view of an hourglass-shaped abdominal aortic aneurysm with two distinct aneurysmal sacs connected by a narrowed segment.

**Fig. 3:**
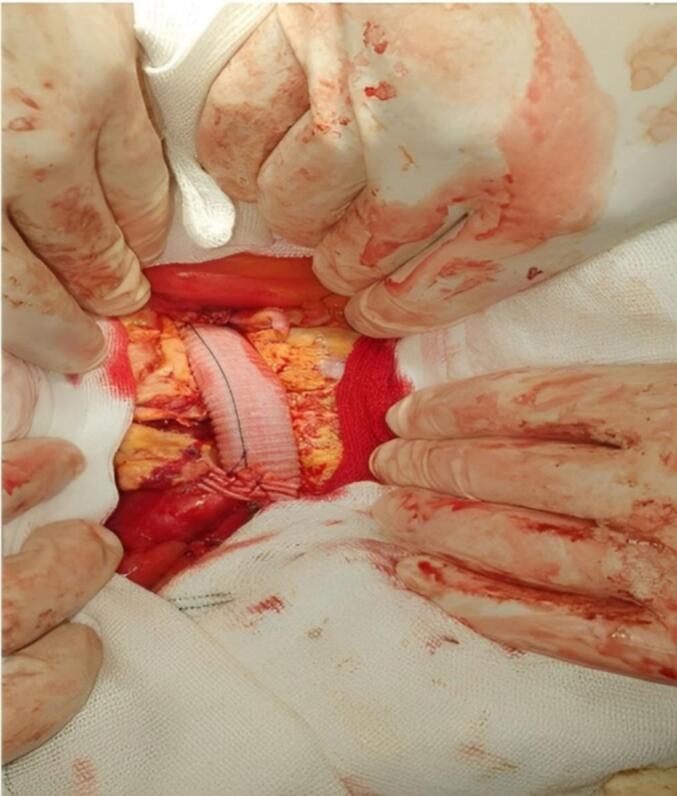
Post-resection view showing successful graft placement following excision of the abdominal aortic aneurysm.

The patient's medical history included diabetic mellitus, hypertension, and a history of cigarette smoking, introducing several risk factors that complicated her clinical management. Given her underlying diabetes and a low ejection fraction of 40 %, the medical team was initially chosen for an endovascular approach to the management of aneurysms, often preferred for high-risk patients due to its minimally aggressive nature and rapid recovery time. However, on further evaluation, it became clear that the proximal landing zone for endovascular repair was insufficient, which inspired us to reevaluate the treatment plan. Eventually, it was decided to proceed with open surgical intervention (Fig. 2, Fig. 3).

The procedure was performed under general anesthesia with laparotomy. Approximately three minutes after the administration of heparin (5000 units IV), distal control was first obtained, followed by proximal control, with total clamp time estimated to be 12–14 min. The aneurysmal segment, including both aneurysms, was excised. A straight Dacron tube graft (approximately 20 mm in diameter) was anastomosed end-to-end using Prolene 3–0 continuous sutures. Hemostasis was confirmed, and pulses were palpable distally. The intraoperative blood loss was minimal (estimated 50–130 mL).

The patient had an uneventful recovery. She was extubated in the operating room, monitored in the ICU for one day, and then transferred to the general ward. She was discharged on postoperative day 4 in stable condition with palpable distal pulses and no signs of ischemia.

Follow-up imaging with Doppler ultrasonography was performed one month post-operation, revealing a patent graft with no residual flow into the excluded aneurysmal sacs. The aneurysms had been completely bypassed, and there were no signs of complications such as thrombosis or pseudoaneurysm formation. The patient remained asymptomatic, and clinical examination confirmed bilaterally normal peripheral pulses.

## 3 Discussion

The management of AAAs involves a choice between open surgery and endovascular approaches, each with its own indications and risks. While the majority of aortic aneurysms present as singular, well-defined dilations, rare cases of dual aneurysms forming an hourglass-shaped configuration have been reported, predominantly in the thoracic aorta (11,12). This case report presents an unusual instance of an hourglass-shaped aortic aneurysm located in the abdominal section of the aorta. Remarkably, this case involves two adjacent abdominal aortic aneurysms that were nearly connected, a presentation scarcely encountered in clinical evidence.

The differential diagnoses considered included a variety of conditions with similar clinical presentations. The patient first presented with chronic abdominal pain, which can be suggestive of a variety of conditions like acute mesenteric ischemia, perforated viscus, and renal colic. The initial ultrasound, followed by CT angiography, helped narrow down the diagnosis to an abdominal aortic aneurysm. While mesenteric ischemia and renal stones were considered due to the patient's risk factors, the imaging findings provided critical evidence in favor of an aneurysm diagnosis. The CT angiogram confirmed the presence of the aneurysm and highlighted its atypical hourglass shape, distinguishing it from other possible causes of abdominal pain.

Double abdominal aortic aneurysms raise important considerations for each condition, its treatment, and surgical approach. Morphologically different hourglass AAAs pose difficulties in decision-making due to the anatomic configuration affecting whether endovascular repair is possible. Although endovascular aneurysm repair is often preferred for high-risk patients due to its minimally invasive nature and shorter recovery times (13), in this present case, the insufficient proximal landing zone rendered EVAR unsuitable, necessitating open surgical intervention. This highlights a critical limitation of endovascular approaches in cases with complex vascular anatomy.

The result of this case effectively illustrates several key points. First, adequate preoperative imaging is paramount to aneurysm morphology description and treatment planning. CT angiography remains the gold standard in assessing aneurysm dimensions, thrombus formation, and the feasibility of stent graft placement. Doppler ultrasonography was initially used for detection, followed by a CT angiogram to assess the full extent and suitability for surgical intervention. Importantly, approximately three minutes after the administration of heparin, distal control was first obtained, followed by proximal control. These critical steps were essential for ensuring hemodynamic stability and maintaining surgical precision. Second, patient-specific factors, such as cardiovascular comorbidities and anatomical constraints, should guide the selection of intervention type. In this instance, despite the patient's comorbid conditions, open repair was deemed the safest and most durable option.

Finally, this case highlights the value of individualized surgical planning in the context of aneurysmal disease complexity. While EVAR has revolutionized the treatment of routine AAAs, open repair is still the mainstay for patients with complex anatomical presentations. The patient's good postoperative result supports the fact that despite its invasiveness, open surgery remains a viable and often necessary approach in select cases.

## 4 Conclusions

In conclusion, hourglass-shaped AAAs represent a rare but clinically significant entity requiring careful diagnostic and therapeutic consideration. The present case contributes to the limited existing literature and reinforces the necessity of personalized management strategies to optimize outcomes in complex aortic aneurysms. Future studies should focus on refining classification criteria for such aneurysms and developing advanced endovascular techniques to expand treatment options for patients with challenging vascular anatomy.

## Informed consent

Written informed consent was obtained from the patient guardian for publication of this case report and accompanying images. A copy of the written consent is available for review by the Editor-in-Chief of this journal on request.

## Consent

Informed written consent was obtained from the patient for publication of this Case report along with any accompanying images. On request, a copy of the written consent is available for review by the Editor-in-Chief of this journal.

## Ethical approval

Ethical approval for this study (IR.TUMS.SINAHOSPITAL.REC.1403.160) was provided by the Ethics Committee of Tehran University of Medical Sciences, Tehran, IRAN.

## Ethics approval

The study was carried out by the Declaration of Helsinki and was registered by the ethics committee of the Department of Cardiology.

## Funding

No funding.

## Author contribution

JS, PP, and AS were responsible for the surgical management of the patient and data acquisition. AS and AF contributed to drafting the manuscript. JS and PP critically reviewed and revised the manuscript for intellectual content. All authors have read and approved the final version of the manuscript and agree to be accountable for all aspects of the work.

## Guarantor

Amir Shokri

## Research Registration Number

Not applicable.

## Conflict of interest statement

The authors declare that they have no competing interests.
